# Pressure-temperature stable conditions of the hydride-ion conductive phase in BaH_2_ and SrH_2_

**DOI:** 10.1080/14686996.2026.2691690

**Published:** 2026-06-23

**Authors:** Satoshi Nakano, Hiroshi Fujihisa, Hiroshi Yamawaki, Yuki Shibazaki, Takumi Kikegawa, Shin-Ichi Orimo

**Affiliations:** a Research Center for Materials Nanoarchitectonics (MANA), National Institute for Materials Science (NIMS), Tsukuba, Ibaraki, Japan; b National Metrology Institute of Japan (NMIJ), National Institute of Advanced Industrial Science and Technology (AIST), Tsukuba, Ibaraki, Japan; c Photon Factory (PF), Institute of Materials Structure Science (IMSS), High Energy Accelerator Research Organization (KEK), Tsukuba, Ibaraki, Japan; d Advanced Institute for Materials Research (WPI-AIMR), Tohoku University, Sendai, Japan; e Institute for Materials Research (IMR), Tohoku University, Sendai, Japan

**Keywords:** Barium hydride, strontium hydride, hydride-ion conduction, high-pressure and high-temperature, phase diagram

## Abstract

The high-pressure (HP)/high-temperature (HT) phase diagram of barium hydride (BaH_2_) was investigated using X-ray diffraction measurements and diamond-anvil cells to clarify the relationships between some phases, including the hydride-ion (H^–^) conductive HT phase that adopts the Ni_2_In-type structure. The results revealed that the HT phase and the HP phase were the same in the phase diagram and constitute an HP/HT phase. Furthermore, it was found that the Ni_2_In-type structure is stable over a wide range up to approximately 50 GPa and 500°C. The coexistence of two phases during the phase transition may be responsible for the previously reported anomalous pressure dependency of ionic conductivity in the HP phase. Additionally, the HP/HT phase diagram of strontium hydride (SrH_2_), which exhibits a similar HP structural sequence, was also investigated. It was found that the Ni_2_In-type structure of SrH_2_ is stable at pressures above 7 GPa within the temperature range of this work (~500 °C). These wide stable HP/HT regions imply that the structural properties of hydride-ion conductive BaH_2_ and SrH_2_ can be investigated under the wide conditions in the future. Furthermore, the pressure dependence of the lattice parameters and volume was obtained, and the bulk moduli of BaH_2_ and SrH_2_ in the Ni_2_In-type structure are determined to be 31.5(15) GPa and 42.4(7) GPa, respectively. The pressure dependence of the crystallographic parameters obtained in this study will be useful for understanding the origin of the properties from the crystallographic viewpoint in future work.

## IMPACT STATEMENT

The stability conditions and crystallographic parameters of the high-pressure phases of BaH_2_ and SrH_2_, which exhibit hydride-ion conductivity, were investigated. The obtained information will contribute to elucidating their conduction mechanism.

## GRAPHICAL ABSTRACT

**Figure:**
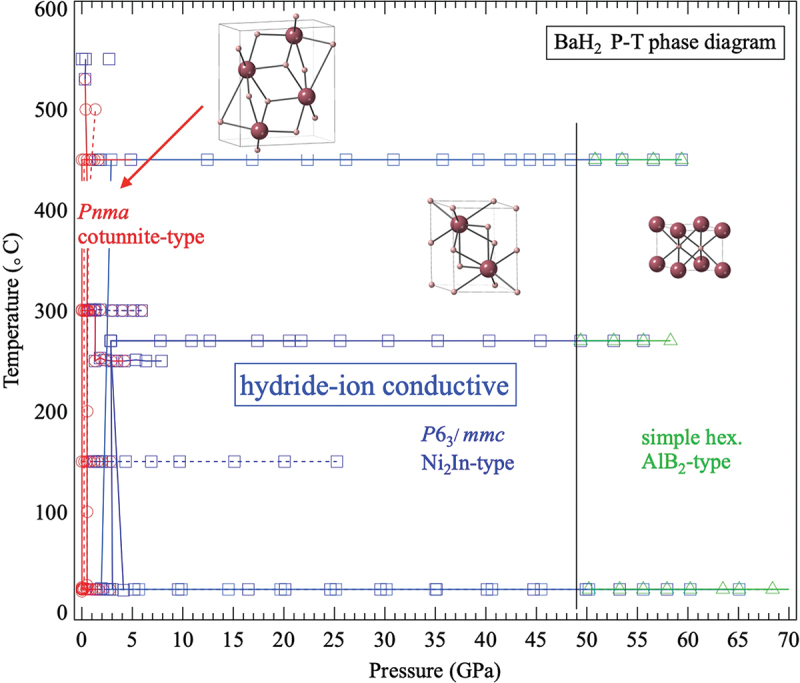

## 1. Introduction

Many researchers have been actively studying the production, transport, and utilization of hydrogen to achieve a sustainable energy society. In materials science, research is underway to develop next-generation energy devices that surpass lithium-ion batteries and hydrogen fuel cells. Ionic conductors used as electrodes and electrolytes, which are the core materials of secondary batteries and fuel cells, have been developed using proton (H^+^) and lithium ion (Li^+^) as carriers. In contrast, recently, ionic conductors that use hydride ion (H^–^), a hydrogen anion, as a carrier have also attracted attention. Hydride ion has an ionic radius similar to that of oxide ion (O^2–^) and fluoride ion (F^–^), making it suitable for ionic conductivity. Furthermore, it has a low redox potential, meaning it has strong reducing power. Therefore, using hydride-ionic conductors may enable the realization of high-potential, high-capacity energy devices. In this background, research on hydride-ion conductors has been actively conducted in recent years, and candidate materials useful as devices have been reported [1–3]. However, the mechanism of hydride-ion conduction is still under investigation.

BaH_2_, a simple binary hydride, adopts a PbCl_2_ (cotunnite)-type structure (*Pnma*) at room temperature (RT) and ambient pressure (AP), and undergoes a phase transition to a high-temperature (HT) phase with a Ni_2_In-type structure (*P*6_3_/*mmc*) at temperatures above approximately 500°C [4,5]. Verbraeken et al. reported that the HT phase of BaH_2_ exhibits high ionic conductivity of 0.2 S cm^−1^ at 630°C [1]. Elucidating the mechanism of hydride-ion conduction in simple binary hydrides like BaH_2_ is important. However, the stable temperature range of the HT phase of BaH_2_ is limited to a narrow range of 500–670°C, limiting the conditions for experimentally investigating its structural properties.

On the other hand, phase transitions in BaH_2_ under high pressure (HP) have also been reported. Kinoshita et al. performed HP X-ray diffraction (XRD) experiments using a diamond-anvil cell (DAC) up to approximately 65 GPa and reported that BaH_2_ undergoes a pressure-induced phase transition from the cotunnite-type to an HP phase with the Ni_2_In-type structure at approximately 2.5 GPa, and further undergoes another phase transition to an AlB_2_-type structure at approximately 50 GPa [6]. Smith et al. conducted XRD and Raman scattering measurements up to approximately 22 GPa and reported the transition pressure to the Ni_2_In-type of 1.6 GPa and the bulk modulus of the phase [7]. Tse et al. performed XRD and Raman scattering measurements up to 58 GPa and reported a phase transition from a Ni_2_In-type structure to a simple hexagonal structure between 40 GPa and 45 GPa [8]. In computational research, Luo et al. performed ab initio electronic structure calculations and reported that the phase transition from cotunnite-type to the Ni_2_In-type structure occurs at 4 GPa [9]. However, since the atomic positions of hydrogen cannot be determined by XRD measurements, it cannot be definitively confirmed that the HP phase adopts a Ni_2_In-type structure. In contrast, Novak et al. performed HP neutron diffraction experiments on BaD_2_ up to 11.3 GPa and confirmed that the crystal structure of the HP phase that appeared in the pressure-induced phase transition between 1.3 and 4.9 GPa is a Ni_2_In-type structure [10]. It is important here that the structure of the first HP phase of BaH_2_ is the same Ni_2_In-type structure as the HT phase. This suggests that the HP phase of BaH_2_ may exhibit ion conduction with hydride-ion carriers similar to the HT phase.

Zhang et al. performed in-situ impedance spectroscopy measurements under HP at RT and determined the diffusion coefficient, grain conductivity, and grain relaxation frequency up to 11.2 GPa [11]. Notably, these values increased significantly in the compression from 2.3 GPa to 6 GPa, after which they continued to increase gradually. To further explore the underlying mechanism, the authors calculated the energy barriers in the H^–^ transport path using density functional theoretical calculations. These results revealed that the HP phase of BaH_2_ with the Ni_2_In-type structure exhibits high ionic conductivity, similar to the HT phase with the same structure. However, the relationship between the HP phase and the HT phase has not been confirmed.

In MH_2_-type alkaline earth metal hydrides, SrH_2_ has the same cotunnite-type structure as BaH_2_ at RT and AP [12]. SrH_2_ has been reported to undergo a phase transition to an HT phase at 856°C under AP [13], however its stable range is limited up to 946°C, and its crystal structure has not been clarified. The ionic conductivity of SrH_2_ has been measured only up to approximately 650°C, and the properties of the HT phase are unknown. On the other hand, under HP, Smith et al. have reported that the HP structural change of SrH_2_ follows a similar structural sequence to that of BaH_2_ [14]. Therefore, the properties of the HP phase of SrH_2_ are also interesting from the perspective of hydride-ionic conduction. In fact, Wang et al. measured the alternating-current (AC) impedance of SrH_2_ up to approximately 25 GPa at RT and reported that a conduction transition from pure electronic to mixed ionic-electronic conduction was observed in relation to the structural phase transition from the cotunnite-type to Ni_2_In-type structure [15].

To further advance the hydride-ion conduction in the Ni_2_In-type phases of BaH_2_ and SrH_2_, measurement of the temperature dependence of ionic conductivity under HP is expected. However, there have been no reports on the stable conditions of these phases under HP/HT. Clarifying the stable pressure-temperature range of the Ni_2_In-type phases and determining their crystallographic parameters is necessary for advancing their conduction properties. Therefore, in this study, the HP/HT phase diagrams of BaH_2_ and SrH_2_ were investigated in the range up to 70 GPa and 550°C using in-situ HP/HT XRD experiments with an externally heated DAC. In addition, the pressure dependence of lattice parameters and volume was examined using a helium pressure medium with high hydrostaticity. The interatomic distance through which the hydride ions pass and the bulk modulus were determined from the crystallographic parameters obtained.

## 2. Experimental

### 2.1. Preparation of the sample and HP cells

The sample powder used was commercially available BaH_2_ and SrH_2_ (purity 99.5%, 60 mesh, Mitsuwa Chemicals Co., Ltd., Japan). All sample handling was performed in an argon-filled glove box with oxygen and water vapor concentrations of 1 ppm or less.

**Figure 1.**
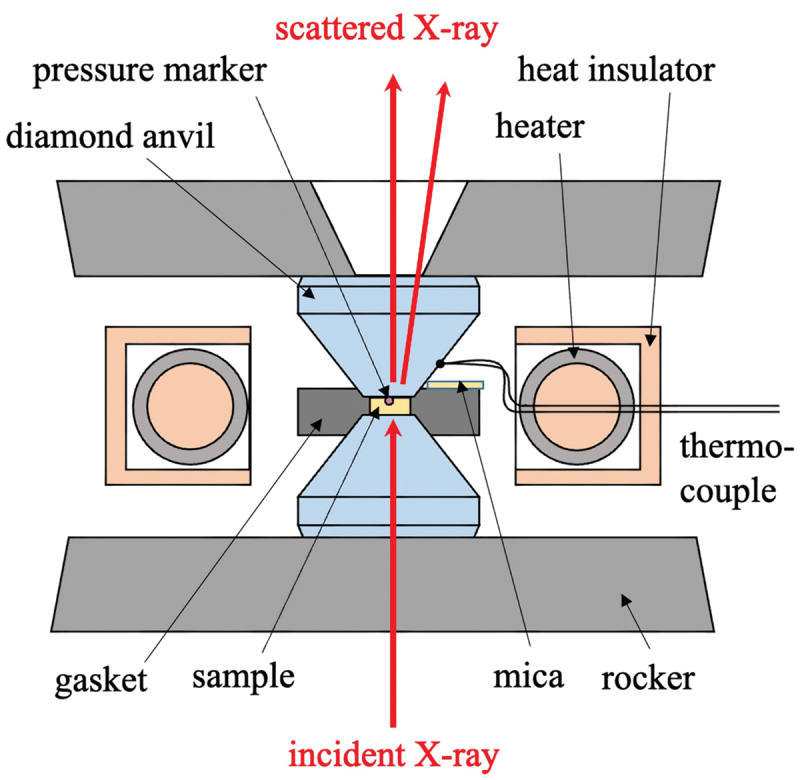
Schematic illustration of the sample area inside the diacell OmniDAC for HP/HT experiments up to 600°C.

A Mao-Bell type DAC combined with a band heater and a Diacell OmniDAC (Almax easyLab, Belgium) equipped with an internal ring heater (see Figure 1) were used for HP/HT experiments up to 300°C and 600°C, respectively. The experiments at RT were performed using a rectangular prism-type DAC with external dimensions of 50 mm × 50 mm × 55 mm. The DACs were equipped with a pair of 1/4-carat diamond anvils with 300–600 μm diameter culets. A rhenium foil, 250–300 μm thick, was pre-indented between the anvils to a thickness of 60–70 μm to serve as a gasket. A 150–300 μm diameter hole was drilled at the center of the gasket using an electrical discharge machine to form the sample chamber together with the anvils.

HP/HT experiments were carried out without a pressure medium, and in some measurements at RT, a helium gas (G1 grade, purity: 99.99995%) as a pressure medium was loaded with the sample using a gas-loading system [16].

As pressure markers, ruby spheres or Sm^2+^-doped yttrium-aluminum garnet (SrB_4_O_7_:Sm^2+^) powder were placed near the center of the anvil culet. The sample pressure was determined from the shift of their fluorescence line [17,18].

The sample temperature was measured using a ceramic-coated K-type thermocouple with a diameter of 0.2 mm cemented to the slope of the anvil. As the diamond anvil exhibits high thermal conductivity, the sample temperature can be assumed to be approximately the same as the anvil temperature.

### 2.2. HP XRD measurements

Angle-dispersive XRD measurements were conducted using synchrotron radiation at the BL-18C and AR-NE1A beamlines of the Photon Factory at the High Energy Accelerator Research Organization (KEK-PF). The X-ray beam was monochromatized to 20 keV at BL-18C and 30 keV at AR-NE1A and introduced to the sample in the DAC through a collimator with a 30–100 µm diameter pinhole. Two-dimensional (2D) diffraction patterns were collected in transmission geometry using flat-panel detectors (Rad-icon 2022 and Rad-icon 1520, Teledyne Rad-Icon Imaging Corp., USA) for room-temperature experiments and an image plate for HT experiments.

The XRD patterns were collected through various pressure-temperature paths to determine the phase boundaries. The 2D diffraction patterns were integrated along the radial direction into one-dimensional (1D) profiles using the image analysis software, IPAnalyzer [19]. Lattice parameters and unit cell volumes were calculated using PDIndexer [19].

The bulk modulus was calculated from the equation of state (EoS), which was obtained by fitting a third-order Birch–Murnaghan equation [20] using the fitting calculation software EoSFit7c [21].

## 3. Results and discussion

### 3.1. Relationship between HT and HP phases of BaH_2_

To clarify the relationship between the HT and HP phases of BaH_2_, the changes in the XRD pattern of BaH_2_ were observed through various pressure-temperature paths in the range of 6 GPa and 550°C.

At RT, the pressure-induced phase transition from the cotunnite-type to the Ni_2_In-type structure occurred at approximately 1.4 GPa. However, the cotunnite-type phase remained even at approximately 2.8 GPa. Kinoshita et al. and Smith et al. both performed HP XRD measurements of BaH_2_ without using a pressure medium, reporting pressure-induced phase transitions at 2.5 GPa and 1.6 GPa, respectively. The present transition pressure is consistent with these previous results. In the present RT experiments, both experiments without a pressure medium and experiments using a helium pressure medium were conducted, but the results were almost identical. It suggests that the pressure-induced phase transition from the cotunnite-type to the Ni_2_In-type structure is sluggish and does not depend heavily on hydrostaticity.

**Figure 2.**
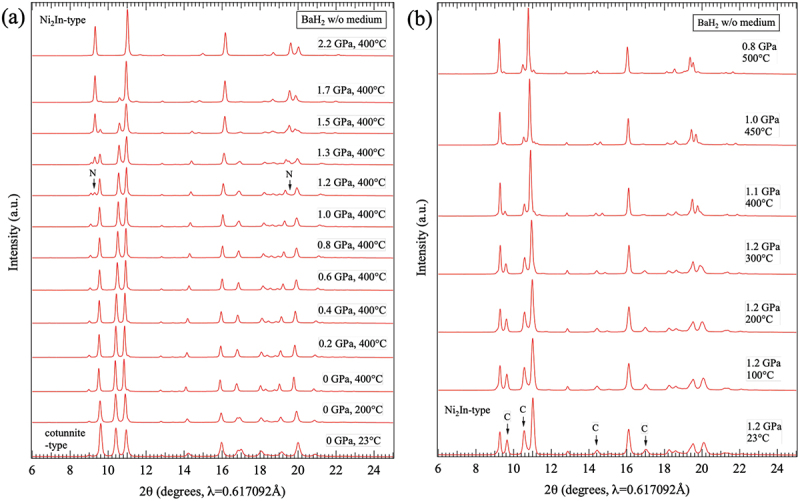
(a) Changes of the XRD patterns when cotunnite-type BaH_2_ was heated to 400°C at AP and then compressed to 2.2 GPa at the temperature. ‘N’ indicates the appearance of the peaks of the Ni_2_In-type structure associated with the pressure-induced phase transition. (b) Changes of the XRD patterns when the two-phase coexistence state of cotunnite-type and Ni_2_In-type BaH_2_ at RT and 1.2 GPa was heated to 500°C. ‘C’ denotes diffraction peaks of the cotunnite-type phase.

Figure 2(a) shows the change in the XRD pattern when BaH_2_ was heated to 400°C at AP and then compressed to 2.2 GPa while maintaining the temperature of 400°C. At the temperature, the peaks corresponding to the Ni_2_In-type structure appeared at 1.2 GPa, and the remaining cotunnite-type phase disappeared at 2.2 GPa. That is, in the pressure-induced phase transition from the cotunnite-type to the Ni_2_In-type, the transition pressure decreases slightly with increasing temperature, and the pressure at which the single-phase Ni_2_In-type is observed also decreases, resulting in a narrowing of the two-phase coexistence region with increasing temperature.

Furthermore, after creating a two-phase coexistence state of cotunnite-type and Ni_2_In-type structures at RT and approximately 1.5 GPa, the pressure was reduced to 1.2 GPa, which is lower than the boundary of 1.4 GPa at RT, and then the temperature was increased. The change of XRD patterns was shown in Figure 2(b). In this case, when the temperature is increased to 100°C, the peak intensity ratio of the cotunnite-type and the Ni_2_In-type hardly changes, or the peak of the cotunnite-type appears to become slightly stronger. However, when the temperature is increased to 200°C or higher, the peak of the Ni_2_In-type clearly becomes stronger, indicating that the pressure-temperature condition has crossed the phase boundary and has entered into the stable conditions for the Ni_2_In-type structure.

**Figure 3.**
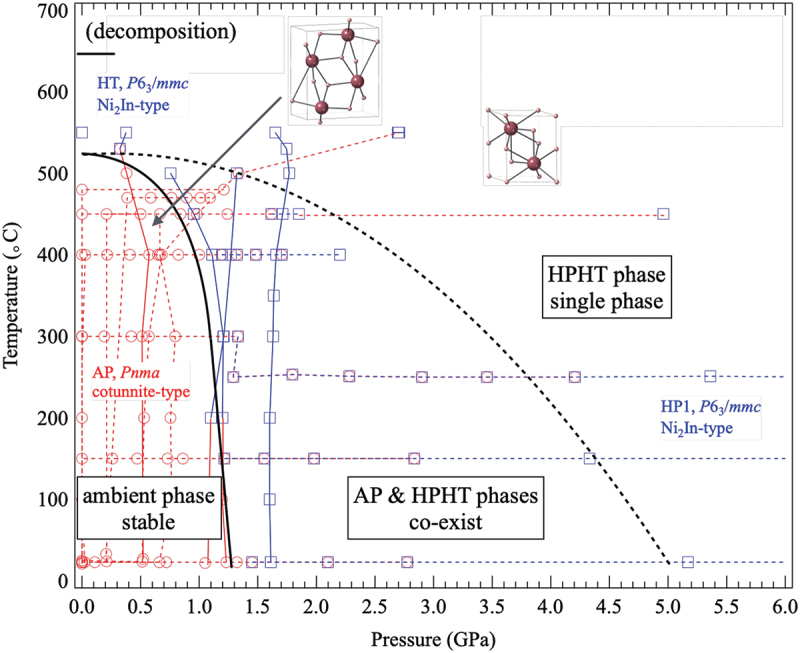
HP/HT phase diagram of BaH_2_ up to 6 GPa and 550°C. All measurements in this phase diagram were performed without the use of a pressure medium. The red dashed line shows the measurement path starting from the cotunnite-type structure. The red circle and the blue square symbols indicate measurement points where the cotunnite-type structure and the Ni_2_In-type structure were observed, respectively. The blue dotted line shows the measurement path where the Ni_2_In-type structure was observed. The blue dotted line shows the measurement path where the Ni_2_In-type structure was observed. The red solid line shows the measurement path starting from the two-phase coexistence state, and the blue solid line shows the measurement path with increased intensity for the Ni_2_In-type rather than the cotunnite-type structure. The black solid line represents the phase boundary between cotunnite-type and Ni_2_In-type phases. The black dashed line indicates the boundary between the Ni_2_In-type single-phase and two-phase coexistence regions.

The results of similar measurements performed under various pressure-temperature paths are summarized in Figure 3 as a pressure-temperature phase diagram. Based on the result, it was found that the boundary between the cotunnite-type phase and the HT phase appearing at approximately 550°C at AP, and the boundary between the cotunnite-type phase and the HP phase, are smoothly connected, indicating that the HT phase and the HP phase are the same phase, i.e. the HP/HT phase. Furthermore, this phase transition is sluggish, and even in the stable conditions of the Ni_2_In-type structure during the compression process, there is a pressure range in which both phases coexist. This phase transition proceeds more rapidly as the temperature increases, and the pressure range in which the two phases coexist narrows.

### 3.2. Relationship between the coexistence of two phases and ionic conductivity

Zhang et al. have reported the pressure dependence of ionic conductivity of BaH_2_ under HP and RT [11]. The conductivity is low immediately after the phase transition from the cotunnite-type to the Ni_2_In-type structure but increases rapidly with pressure up to about 6 GPa (an increase of about two orders of magnitude in S cm^−1^ units). The authors have explained the rapid increase in conductivity as the ion transfer barrier energy decreases with pressure. In our results, the pressure region where the conductivity increases significantly corresponds to the conditions where the coexistence of the cotunnite-type and the Ni_2_In-type structure is transforming to a single phase of the Ni_2_In-type structure. Furthermore, above 6 GPa, where the single-phase Ni_2_In-type structure is formed, the ionic conductivity continues to increase gradually with pressure, but the rate of increase is not very high. Therefore, the ion transport barrier pointed out by Zhang et al. may be due to the existence of the cotunnite-type phase that remains untransformed.

Incidentally, when extrapolating the pressure dependence of the ionic conductivity of the Ni_2_In-type HP phase measured by Zhang et al. at RT and 6–12 GPa to the lower pressure side, it yields approximately 3 × 10^−6^ S cm^−1^ at AP. On the other hand, when extrapolating the temperature dependence of the ionic conductivity of the Ni_2_In-type HT phase measured by Verbraeken et al. at AP to the lower pressure side, it also yields approximately 3 × 10^−6^ S cm^−1^ around RT. These may be mere coincidences, but they might indicate that the HT phase and the HP phase are the same phase.

### 3.3. Stable pressure and temperature conditions for hydride-ion conductive Ni_2_In-type BaH_2_

The Ni_2_In-type BaH_2_ has been reported to undergo a pressure-induced phase transition to the AlB_2_-type structure at approximately 50 GPa at RT [6]. In this study, the experimental conditions for HP/HT XRD measurements were extended to the higher pressure side to determine the pressure-temperature region in which the hydride-ion conductive BaH_2_ with Ni_2_In-type structure exists stably.

**Figure 4.**
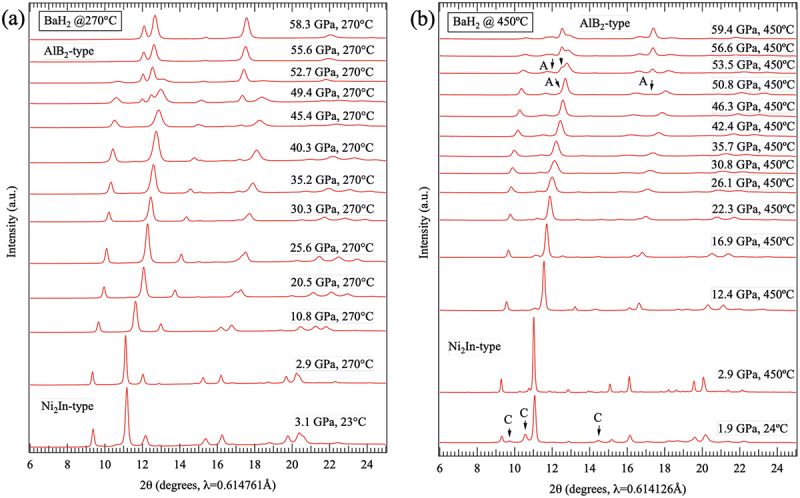
Pressure dependences of the XRD patterns when Ni_2_In-type BaH_2_ was heated (a) to 270°C and (b) to 450°C at 3.1 GPa and then compressed to approximately 59 GPa at the same temperature. ‘C’ and ‘N’ indicate the peaks of the cotunnite-type structure and the Ni_2_In-type structure, respectively. The pressure-induced phase transition from Ni_2_In-type to AlB_2_-type structure occurred at approximately 50 GPa.

Figure 4(a) shows the change in the XRD patterns of the Ni_2_In-type structure in the compression above approximately 3 GPa at 270°C. As a result, the diffraction peak of the AlB_2_-type structure appeared at approximately 50 GPa, almost the same pressure as when compressed at RT. Similar experiments were also carried out at 450°C, and again, the phase transition to the AlB_2_-type structure occurred at approximately 50 GPa, as shown in Figure 4(b).

**Figure 5.**
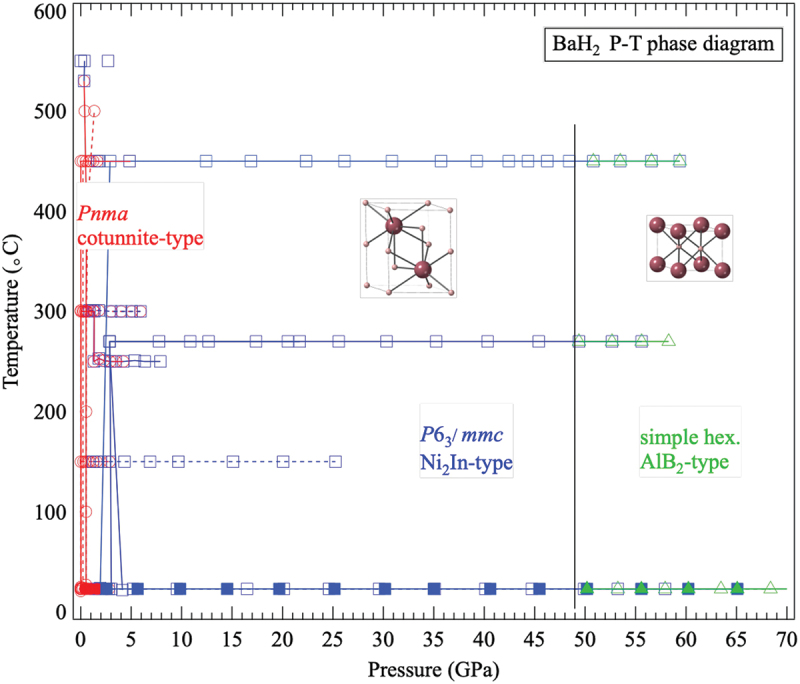
HP/HT phase diagram of BaH_2_ up to 70 GPa and 550°C. The red, blue, and green symbols represent the conditions where cotunnite-type, Ni_2_In-type, and AlB_2_-type structures were observed, respectively. Furthermore, the open and solid symbols represent experiments without a pressure medium and experiments using helium as the pressure medium, respectively. The black solid line represents the phase boundary between Ni_2_In-type and AlB_2_-type phases.

The extended HP/HT phase diagram obtained from these results is shown in Figure 5. It was found that Ni_2_In-type BaH_2_ exists stably in the range of 1.4–50 GPa at RT and is also widely stable from AP to approximately 50 GPa even at HT around 500°C. Zhang et al. showed that the ionic conductivity of the phase continued to increase slightly with increasing pressure up to approximately 11.2 GPa [11]. It is interesting whether conductivity continues to rise up to the upper pressure limit around 50 GPa. If not, the relationship between the conduction nature and the crystallographic parameters may provide important information to elucidate the mechanism of the hydride-ion conduction in alkaline earth metal hydrides. Furthermore, since the Ni_2_In-type structure is stable at least up to the temperature of 450°C, it is expected that the temperature dependence of the ionic conductivity over a wide pressure range will lead to a deeper understanding of the conduction mechanism.

### 3.4. HP/HT phase diagram of SrH_2_

A pressure-induced phase transition of SrH_2_ from the cotunnite-type to the Ni_2_In-type structure, similar to BaH_2_, has been experimentally observed, and further phase transition to an AlB_2_-type structure has been theoretically demonstrated by Smith et al. [14]. This similarity in the HP structural sequences of SrH_2_ and BaH_2_ suggests that SrH_2_ may exhibit similar physical properties to BaH_2_. Indeed, Wang et al. performed AC impedance measurements of SrH_2_ up to approximately 25 GPa at RT and reported that the phase transition from the cotunnite-type to the Ni_2_In-type structure induced the change in conduction carriers from pure electron conduction to mixed-ionic electron conduction [15].

**Figure 6.**
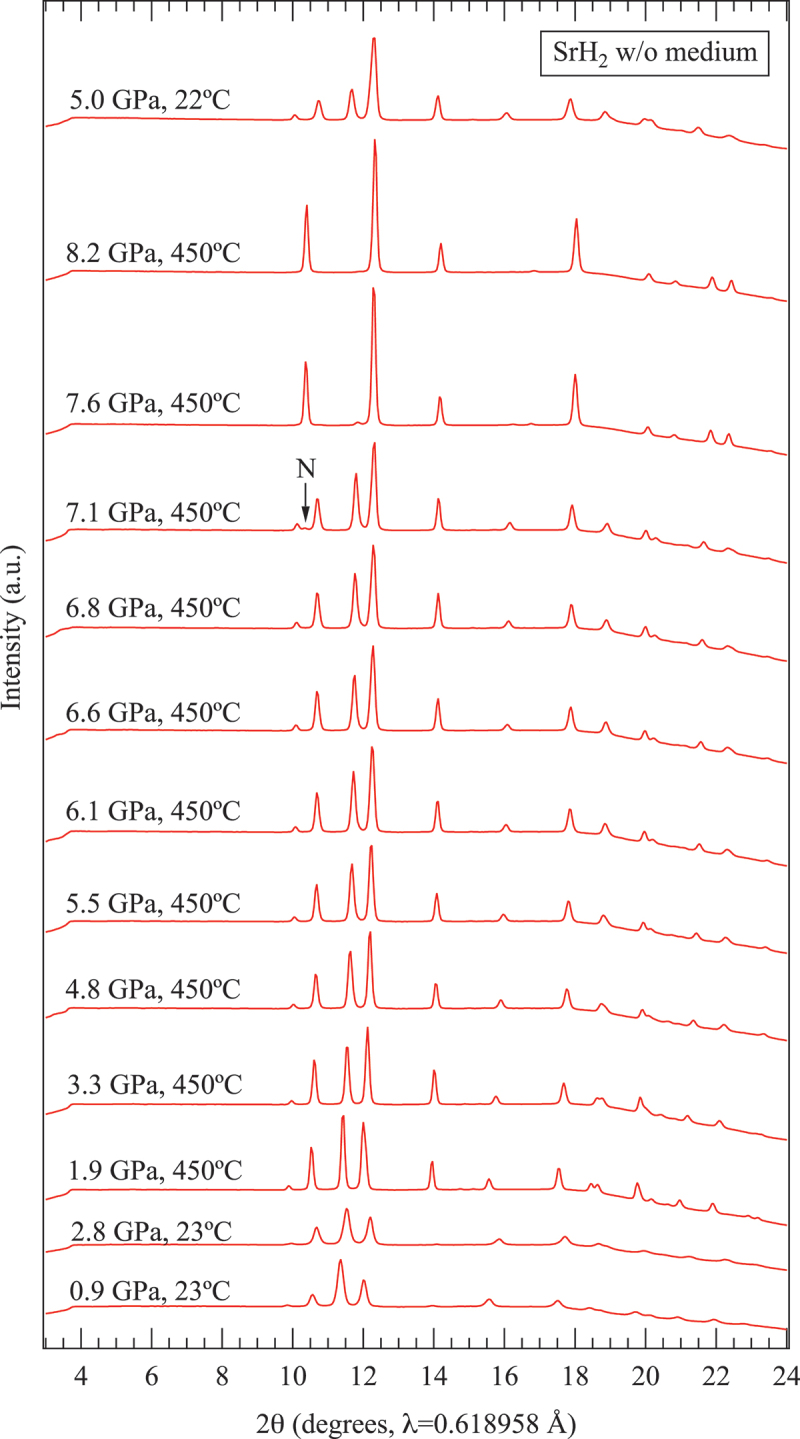
Changes of the XRD patterns when cotunnite-type SrH_2_ was compressed to 2.8 GPa, heated to 450°C, and then compressed to 8.2 GPa at the same temperature. ‘N’ indicates the appearance of the peaks of the Ni_2_In-type structure associated with the pressure-induced phase transition.

**Figure 7.**
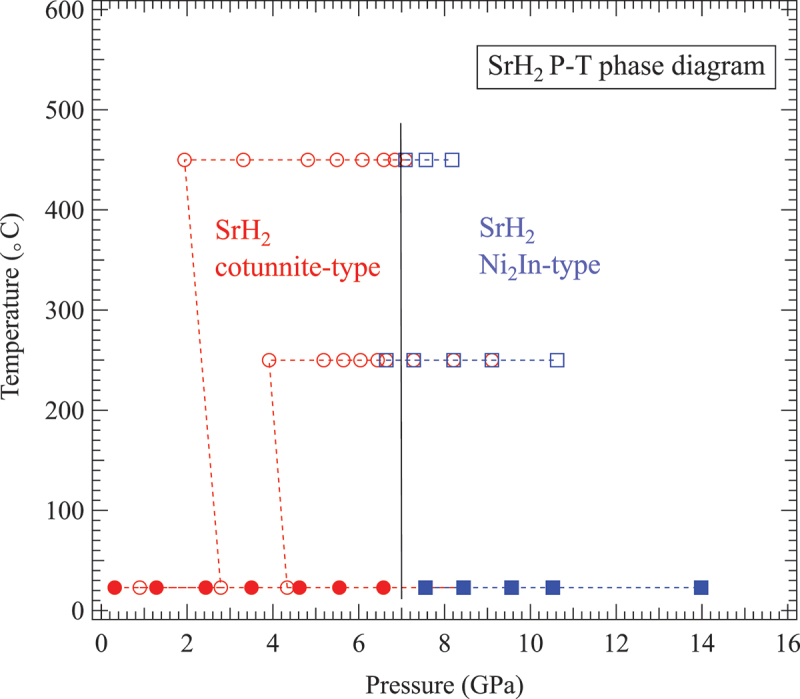
HP/HT phase diagram of SrH_2_ up to 14 GPa and 450°C. The red dashed line shows the measurement path starting from the cotunnite-type structure. The red circle and the blue square symbols indicate measurement points where the cotunnite-type structure and the Ni_2_In-type structure were observed, respectively. Furthermore, the open and solid symbols represent experiments without a pressure medium and experiments using helium as the pressure medium, respectively. The blue dotted line shows the measurement path where the Ni_2_In-type structure was observed. The black solid line represents the phase boundary between cotunnite-type and Ni_2_In-type phases.

Therefore, to investigate the stability conditions of the Ni_2_In-type structure in SrH_2_, HP/HT XRD measurements similar to those for BaH_2_ were performed in the range of approximately 14 GPa and 450°C. The pressure dependence of the XRD pattern at 450°C is shown in Figure 6, and the obtained HPHT phase diagram is shown in Figure 7. The phase boundary between the cotunnite-type and the Ni_2_In-type structure appears to stand almost perpendicular to the pressure axis in the phase diagram at approximately 7 GPa, regardless of temperature. This small temperature dependence of the phase boundary between the cotunnite-type and Ni_2_In-type structures is similar to that of BaH_2_. Though it has been reported that SrH_2_ undergoes a phase transition to the HT phase β-SrH_2_ at approximately 856°C under AP, its structure is not yet clear [13]. Therefore, the relationship between the HT and HP phases in SrH_2_ has not been clarified.

Smith et al. calculated that SrH_2_ undergoes a phase transition from the Ni_2_In-type to the AlB_2_-type structure at approximately 115 GPa [14]. Therefore, SrH_2_ with the Ni_2_In-type structure is stable over a wider pressure range than BaH_2_. Investigation of a pressure-temperature phase diagram for SrH_2_ up to its upper pressure limit will be a subject for future work.

### 3.5. Pressure dependence of lattice parameters and volume of Ni_2_In-type BaH_2_ and SrH_2_

In general, to investigate the structural properties under HP, accurate measurements of the crystallographic parameters, such as lattice parameters, are important. Regarding the pressure values for the pressure-induced phase transitions of BaH_2_ and SrH_2_, they are not highly dependent on hydrostatic conditions, as confirmed in this study. However, for measurements of crystallographic parameters, experiments in a high-hydrostatic environment are crucial. When applying uniaxial compression using a DAC and measuring diffracted X-rays in the direction of compression, the crystallographic parameters obtained from the XRD patterns are generally affected by hydrostatic pressure. In particular, measurements under low hydrostatic conditions without using a pressure medium tend to yield larger lattice parameters than the average value of the entire sample [22,23]. Therefore, it is desirable to determine crystallographic parameters under high hydrostatic conditions. In this study, the pressure dependence of the lattice parameters of Ni_2_In-type structure BaH_2_ and SrH_2_ was examined from XRD measurements at RT using a helium pressure medium.

**Figure 8.**
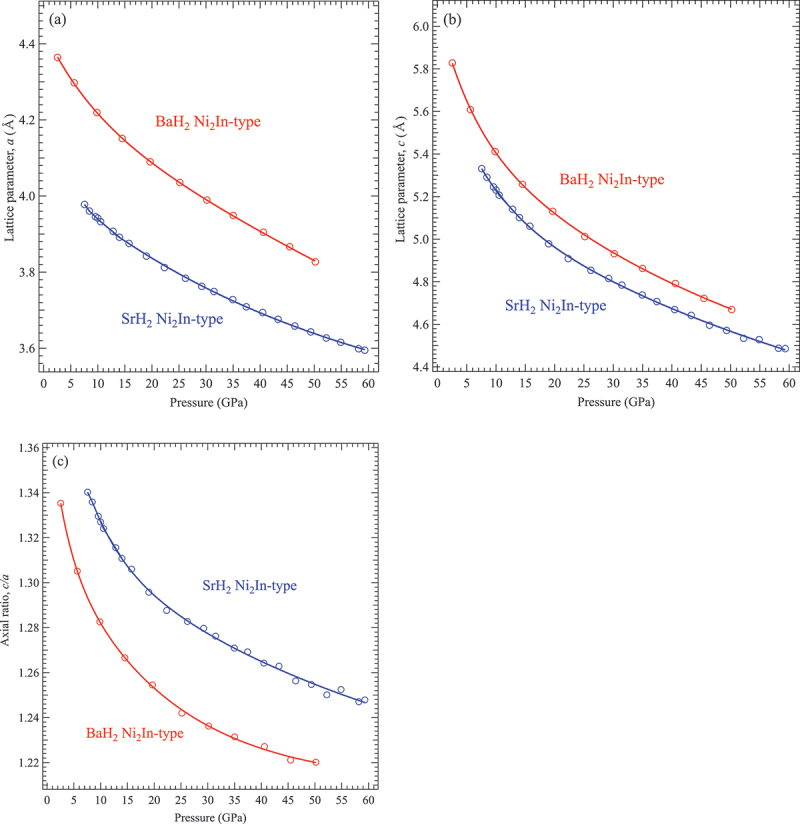
Pressure dependence of the lattice parameters (a) *a*, (b) *c*, and (c) *c/a* axial ratio of Ni_2_In-type BaH_2_ and SrH_2_ in compression. The red and blue symbols represent BaH_2_ and SrH_2_, respectively. The error bars for each data point are smaller than the size of the symbols in the figure.

Figure 8 shows the pressure dependence of the lattice parameters and axial ratio *c/a* for BaH_2_ up to approximately 50 GPa and for SrH_2_ up to approximately 60 GPa. In the Ni_2_In-type structure immediately after the phase transition from the cotunnite-type structure, both the *a*-axis and *c*-axis of BaH_2_ are approximately 10% larger than those of SrH_2_, and the axial ratio *c/a* is the same at approximately 1.34 in both cases, although the pressures are different. At approximately 50 GPa, when BaH_2_ undergoes a phase transition to the AlB_2_-type structure, the axial ratio *c/a* is approximately 1.22, while the axial ratio of SrH_2_ is only about 1.25 even at approximately 60 GPa. Extrapolating the *c/a* pressure-dependent curve for SrH_2_ in Figure 8, it appears that the *c/a* ratio reaches approximately 1.22 around 115 GPa, at which Smith et al. predicted a phase transition from the Ni_2_In-type to the AlB_2_-type structure in SrH_2_ occurs [14].

**Figure 9.**
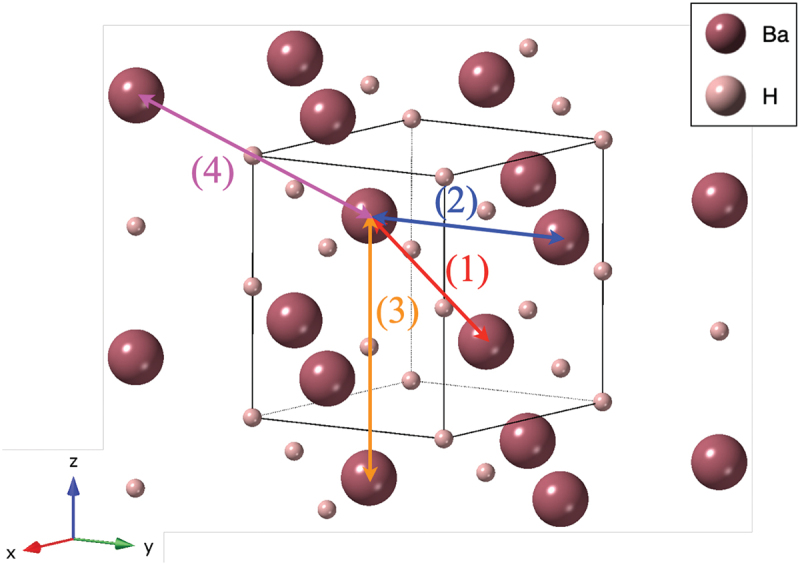
The crystal structure of Ni_2_In-type BaH_2_ and four types of the interatomic distances of the barium ions. The burgundy and pink balls indicate barium and hydrogen atoms, respectively.

In hydride-ion conduction of MH_2_-type hydrides, hydride ions move between metal cations. It is useful to determine the interatomic (M–M) distances at various pressures from crystallographic parameters when investigating the structural properties under HP. Figure 9 shows four types of M–M distance in the crystal structure of Ni_2_In-type BaH_2_, and Table 1 shows the respective M–M distances calculated with the lattice parameters. Zhang et al. [11] and Wang et al. [15] proposed the H^–^ migration paths in the cotunnite and Ni_2_In-type structures of BaH_2_ and SrH_2_, respectively, and calculated the pressure dependences of their energy barriers. The H^–^ migration path in the Ni_2_In-type structure was described as a line joining three H^–^ atoms oriented along the *c*-axis of the hexagonal unit cell, which corresponds to the path across the Ba–Ba or Sr–Sr distance (4) in Table 1. At relatively low pressures, the distances (3) and (4) do not differ considerably; nevertheless, at higher pressures, distance (4) appears to be longer than distance (3). Therefore, when the pressure rises, the H^–^ migration path that Zhang et al. and Wang et al. assumed might become more likely. Recently, LaH_3 − 2x_O_x_ (x < 0.25) [24] and La_1-x_Sr_x_H_3-x-2y_O_y_ (0.1 ≤ x ≤ 0.6, y ≤ 0.171) [25] have been reported to exhibit high H^–^ conductivity around RT. These materials exhibit high H^–^ conductivity by doping the hydrides with oxygen or other cations; their conduction pathways are believed to be more intricate than those of basic binary hydrides like BaH_2_ and SrH_2_. Especially for LaH_3 − 2x_O_x_, molecular dynamics simulations have revealed the presence of both mobile and immobile H^–^. For reference only, therefore, the La–La distances for LaH_3 − 2x_O_x_ are 2.0, 2.8, 3.5, and 4.0 Å, whereas the La(Sr)–La(Sr) distances for La_1-x_Sr_x_H_3-x-2y_Oy are 4.0 and 5.7 Å.

**Table 1.** Interatomic distances of cations in Ni_2_In-type structure BaH_2_ and SrH_2_. The distances (1) to (4) correspond to the interatomic distances shown in Figure 9.

		BaH_2_		SrH_2_	
Pressure (GPa)		2.55	50.2	7.55	59.3
Distance (Å)	(1)	3.852	3.215	3.518	3.056
	(2)	4.364	3.827	3.978	3.595
	(3)	5.828	4.670	5.311	4.486
	(4)	5.821	4.998	5.331	4.718

**Figure 10.**
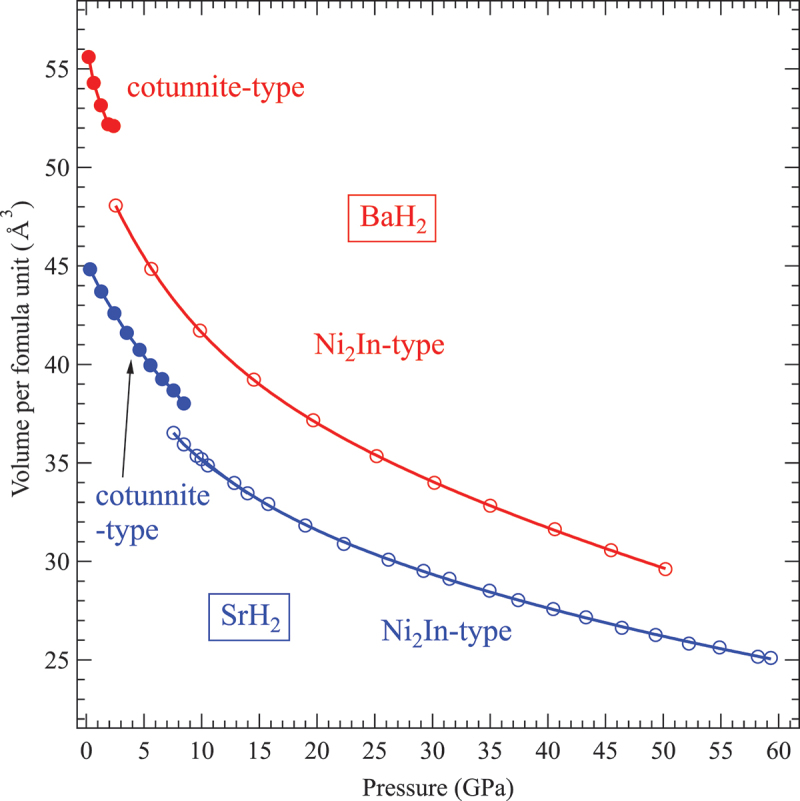
Pressure dependence of the volumes per formula unit of BaH_2_ and SrH_2_ in compression. The solid and open circles represent cotunnite-type and Ni_2_In-type structures, respectively. The error bars for each data point are smaller than the size of the symbols in the figure.

Figure 10 shows the pressure dependence of the volume per unit formula obtained from lattice parameters. From the EoS curves, the bulk modulus of Ni_2_In-type BaH_2_ and SrH_2_ was calculated to be K_0_ = 31.5(15) GPa (V_0_ = 51.4(2) Å^3^, K_0_′ = 3.97(11)) and K_0_ = 41.57(13) GPa (V_0_ = 42.4(7) Å^3^, K_0_′ = 4.01(2)), respectively. Smith et al. determined the bulk modulus of Ni_2_In-type BaH_2_ and SrH_2_ from first-principles calculations and experimental data [7,14]. Luo et al. also determined the bulk modulus from *ab initio* electronic structure calculations [9]. A comparison of these results with those of this study is shown in Tables 2 and 3. The K_0_, V_0_, and K_0_′ obtained in this study agree very well with the theoretical values from first-principles calculations. The present experiments were carried out under high hydrostatic conditions using a helium pressure medium, which might allow for the accurate decision of the pressure dependence of the volume under conditions close to an ideal environment.

**Table 2.** Comparison of the bulk modulus of Ni_2_In-type BaH_2_ obtained in this study with previous studies.

	Smith et al. [7]		Luo et al. [9]	This work
Method	theory	experiment	theory	experiment
V_0_/Z (A^3^)	51.9(1)	54.0(1)	51.62	51.4(2)
K_0_ (GPa)	31.1(2)	24(1)	34.7	31.5(15)
K_0_′	4.13(3)	4.13	3.6	3.97(11)

**Table 3.** Comparison of the bulk modulus of Ni_2_In-type SrH_2_ obtained in this study with previous studies.

	Smith et al. [14]		This work
Method	theory	experiment	experiment
V_0_/Z (A^3^)	40.72(3)	37.8(7)	41.57(13)
K_0_ (GPa)	41.3(2)	75.6(6)	42.4(7)
K_0_′	3.87(1)	3.87*	4.01(2)

*Experimental K_0_’ by Smith et al. was the value based on their theoretical results.

In future work for investigating the properties of these hydride-ion conductive phases, ionic conductivity should be measured under a wide range of temperature and pressure conditions. We believe that the pressure dependence of the crystallographic parameters obtained in this study will be useful for understanding the origin of the properties from the crystallographic viewpoint.

## 4. Conclusion

The HP/HT phase diagram of BaH_2_ was investigated using XRD measurements with DACs in the range up to 70 GPa and 550°C. The results showed that the HT phase and the HP phase were the same HP/HT phase with a Ni_2_In-type structure. Furthermore, this Ni_2_In-type BaH_2_, exhibiting hydride-ion conduction, is stable across the wide pressure range from 1.4 GPa to 50 GPa at RT and from AP to 50 GPa at approximately 500°C. It has been found that the anomalous pressure dependence of ionic conductivity in the HP phase, which has been reported by Zhang et al. previously, may be due to the coexistence of two phases during the phase transition from the cotunnite-type to the Ni_2_In-type structure. In addition, the HP/HT phase diagram of SrH_2_ was also investigated, and the Ni_2_In-type structure, which has also been reported to exhibit hydride-ion conduction, is stable at the pressure range from 7 GPa to at least 60 GPa, and the temperature from RT to approximately 500°C around 7–9 GPa. These wide ranges of stable conditions of Ni_2_In-type BaH_2_ and SrH_2_ suggest that the properties of these hydride-ion conductors can be investigated under a wide range of conditions in the future. In addition, the pressure dependence of the lattice parameters and volume of BaH_2_ and SrH_2_ with Ni_2_In-type structures at RT revealed that their bulk moduli are 31.5(15) GPa and 42.4(7) GPa, respectively. The pressure dependence of the crystallographic parameters obtained in this study will be useful for understanding the origin of the properties from the crystallographic viewpoint in future work.

## Supplementary Material

Supplemental Material
