# Supplementary material for: Pressure-temperature stable conditions of the hydride-ion conductive phase in BaH2 and SrH2
Source: Sci Technol Adv Mater. 2026 Jun 23;27(1):2691690. doi: 10.1080/14686996.2026.2691690 (PMC13348106; doi:10.1080/14686996.2026.2691690)
Supplement: Supplemental Material [file TSTA_A_2691690_SM0610.pdf]

supplementary material

Pressure-temperature stable conditions of the hydride-ion conductive phase in BaH_2 and SrH_2

Satoshi Nakano [a]* Hiroshi Fujihisa[b] Hiroshi Yamawaki[b] Yuki Shibazaki[c] Takumi Kikegawa[c] Shin-ichi Orimo[d,e]

[a]*Research Center for Materials Nanoarchitectonics (MANA), National Institute for Materials Science (NIMS), Tsukuba, Ibaraki, Japan;* [b]*National Metrology Institute of Japan (NMIJ), National Institute of Advanced Industrial Science and Technology (AIST), Tsukuba, Ibaraki, Japan;* [c]*Photon Factory (PF), Institute of Materials Structure Science (IMSS), High Energy Accelerator Research Organization (KEK), Tsukuba, Ibaraki, Japan;* [d]*Advanced Institute for Materials Research (WPI-AIMR), Tohoku University, Sendai, Miyagi, Japan;* [e]*Institute for Materials Research (IMR), Tohoku University, Sendai, Miyagi, Japan*

* Corresponding author: Satoshi Nakano, e-mail: NAKANO.Satoshi@nims.go.jp, address: National Institute for Materials Science (NIMS), Tsukuba, Ibaraki 305-0044, Japan

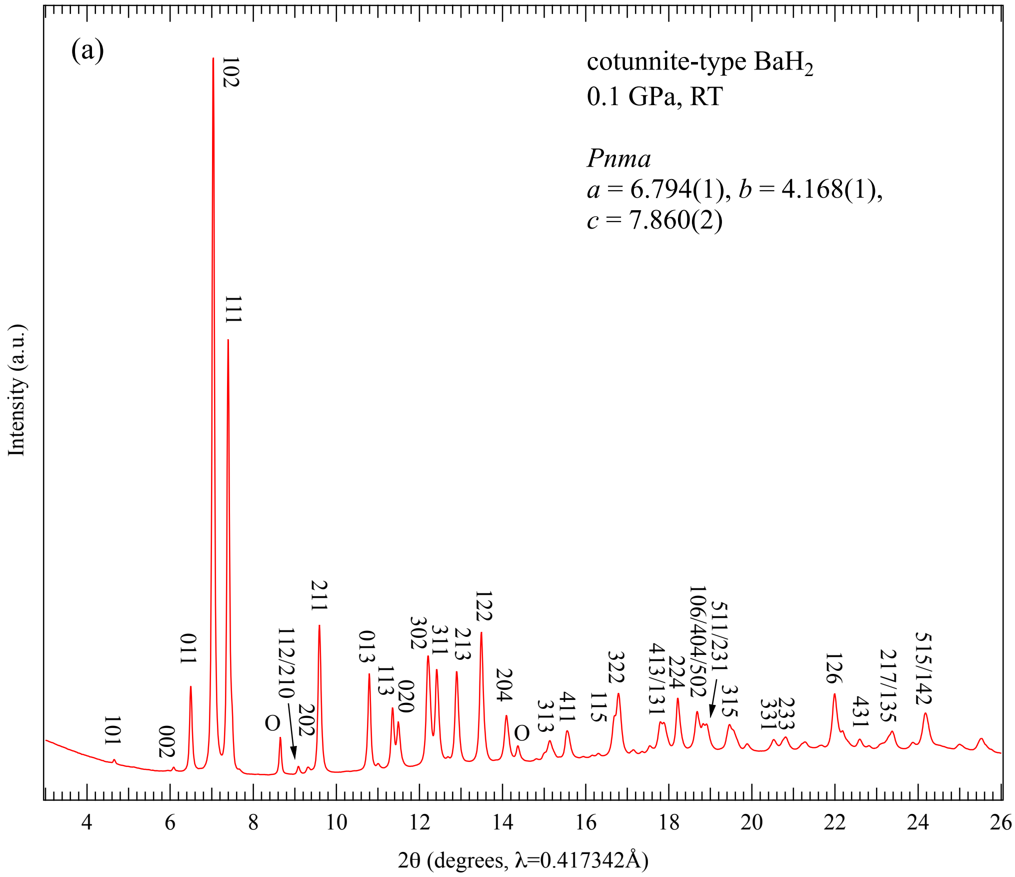

(a)
cotunnite-type BaH2
0.1 GPa, RT
Pnma
a = 6.794(1), b = 4.168(1),
c = 7.860(2)
Intensity (a.u.)
2θ (degrees, λ=0.417342Å)

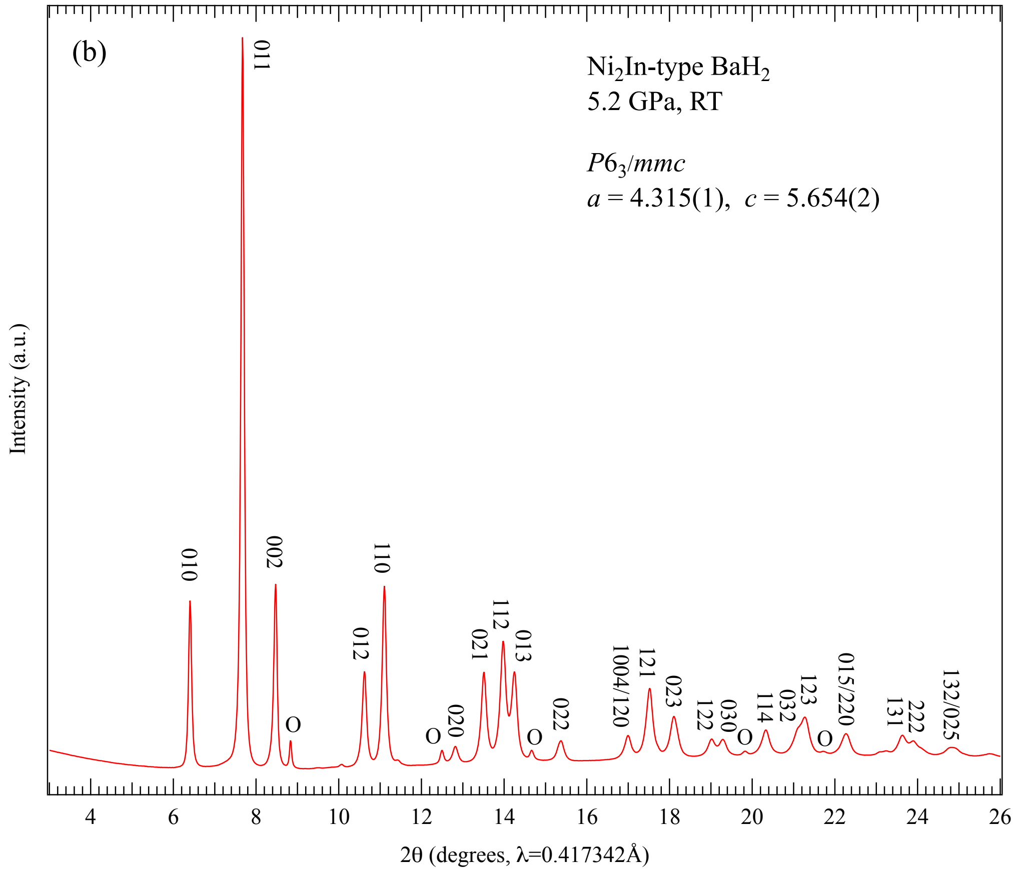

(b)
Ni2In-type BaH2
5.2 GPa, RT
P63/mmc
a = 4.315(1), c = 5.654(2)
Intensity (a.u.)
2θ (degrees, λ=0.417342Å)

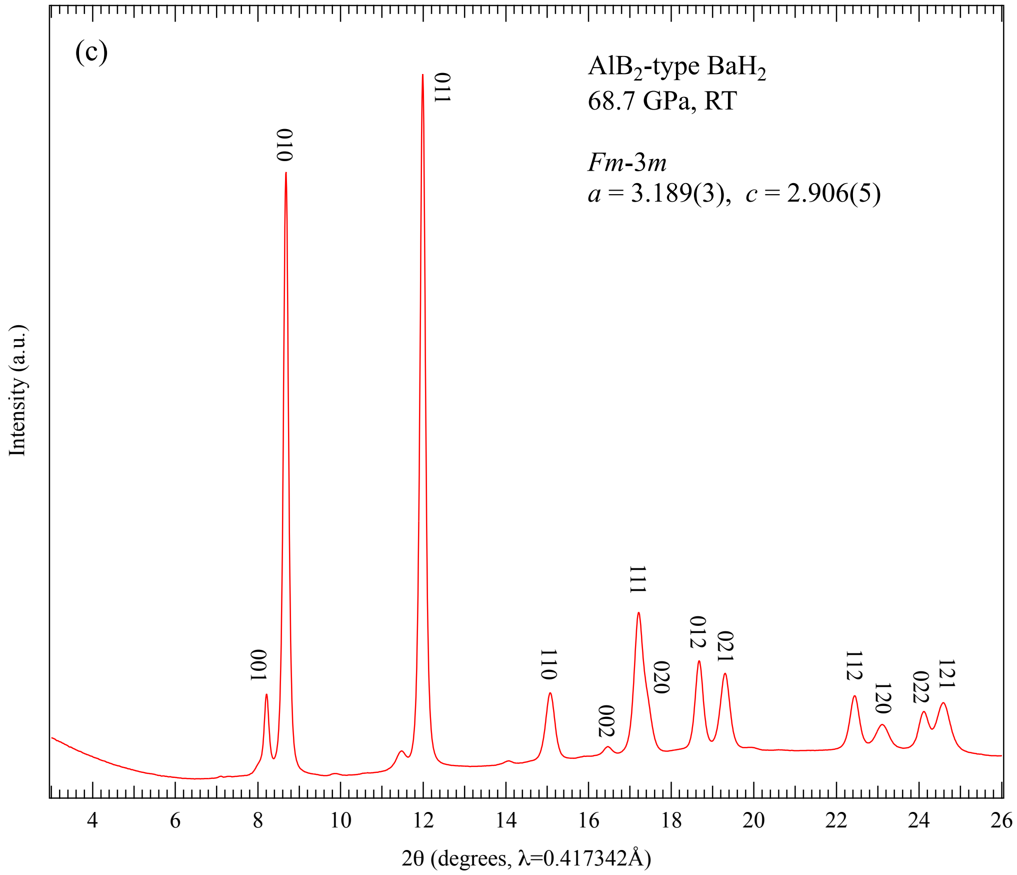

Figure S1. X-ray diffraction (XRD) pattern of BaH_2 with (a) the cotunnite-type structure obtained at 0.1 GPa, (b) Ni_2In-type structure obtained at 5.2 GPa, and (c) AlB_2-type structure obtained at 68.7 GPa and room temperature (RT). The three-digit number indicates the Miller index of the diffraction peak. "O" denotes diffraction peaks of MgO contamination.
